# A Novel Assessment of Infratubercle Sagittal Proximal Tibial Morphology and Relationship to Full Length Posterior Tibial Slope: Lateral Infratubercle Angle

**DOI:** 10.1177/23259671261445953

**Published:** 2026-06-05

**Authors:** Alfred Mansour, Jessica Dziuba Waters, Nicole Lemaster, Michael Kutzler, Connor Fritz, Walter Lowe, Alexis Aboulafia

**Affiliations:** † Department of Orthopaedic Surgery, The University of Texas Health Center, Houston, Texas, USA; ‡ Memorial Hermann Rockets Sports Medicine Institute, Houston, Texas, USA; Investigation performed at the Department of Orthopaedic Surgery, The University of Texas Health Center at Houston, Houston, Texas

**Keywords:** high tibial osteotomy, posterior tibial slope, slope-reducing osteotomy

## Abstract

**Background:**

Slope-reducing high tibial osteotomy (SRO-HTO) surgically corrects elevated posterior tibial slope (PTS) and is often performed in the setting of failed anterior cruciate ligament (ACL) reconstruction. Lateral supratubercle angle (LSTA) quantifies the supratubercle component of elevated PTS, but the infratubercle component of elevated PTS has not been quantified.

**Hypothesis/Purpose:**

The purpose of this study was to describe and establish mean values for lateral infratubercle angle (LITA) within non-ACL tear, primary ACL tear, and ACL graft tear cohorts. It was hypothesized that LITA would differ between non-ACL tear and ACL graft tear cohorts, would positively correlate with PTS elevation, and, when used with LSTA, would localize the deformity leading to PTS elevation.

**Study Design:**

Cross-sectional study; Level of evidence, 3.

**Methods:**

Patients seen for a knee complaint were categorized into non-ACL tear, primary ACL tear, or ACL graft tear cohorts. PTS, LSTA, and LITA were measured on full-length lateral (FLL) tibia radiographs. Descriptive statistics were calculated for each measurement and cohort, and comparisons were performed using a 1-way analysis of variance with a Bonferroni correction (*P* < .01 indicating statistical significance). The non-ACL tear cohort's mean LITA value served as the cutoff. Pearson's correlation determined the relationship between elevated PTS with LITA and LSTA. *P* < .05 indicated statistical significance.

**Results:**

A total of 300 patients were included, 100 per cohort. The mean LITA in the non-ACL tear, primary ACL tear, and ACL graft tear cohorts were 89°± 4.6°, 90.7°± 5.8°, and 92°± 5°, respectively. Significant differences existed in LITA between the non-ACL tear and ACL graft tear cohorts (*P* < .001). Within the ACL graft tear cohort, 74.4% (64/86) of patients with elevated medial or lateral PTS (PTS-M/L) had elevated LITA, regardless of LSTA elevation status. Significant positive correlations existed between PTS-M/L and LITA in the non-ACL tear (*P* = .011/*P* = .004), primary ACL tear (*P* = .002/*P* = .029), and ACL graft tear cohorts (*P* < .001/*P* < .001/*P* < .001).

**Conclusion:**

LITA is a novel measurement, with distinct anatomic landmarks, that quantifies infratubercle deformity and can be easily incorporated into FLL radiographic assessment. Elevated LITA was present in most patients with elevated PTS in both the primary ACL tear and ACL graft tear cohorts, indicating a contribution from infratubercle deformity. We recommend utilizing LITA and LSTA with PTS to quantify region-specific proximal sagittal deformity. Future studies should determine whether this quantification can help surgeons select the SRO-HTO technique and influence outcomes.

It has been well established that an elevated posterior tibial slope (PTS) is an independent risk factor for graft failure after anterior cruciate ligament (ACL) reconstruction and increases anterior tibial translation by placing greater shear force on both native and graft anterior cruciate ligaments.^2,4,11,15,21,26,29,36^ Slope-reducing high tibial osteotomy (SRO-HTO) is the preferred surgical correction for elevated PTS and has been shown to decrease ACL graft failure and improve knee biomechanics.^3,7,9,10,11,12,30,31,35^ Several surgical techniques for elevated PTS correction have been published, with 3 primary levels of correction: proximal to the tibial tubercle (supratubercle), at the tubercle (transtubercle), and distal to the tubercle (infratubercle).^6,12,28,32,35^ The surgical technique utilized is often based on surgeon preference and not on the level-specific morphology of the proximal tibia deformity, with supratubercle HTO-SRO being utilized often.^12,28,32,35^ This can be partially attributed to the inability of current PTS measurement techniques to locate the proximal tibia deformity in relation to the tibial tubercle.

There are several methods for measuring PTS; however, there is no consensus on which technique and modality most accurately assess the deformity.^1,18,26,31-33^ Furthermore, without the ability to characterize the morphology of proximal tibial deformity, it is challenging to tailor the surgical osteotomy technique to ensure the primary contributor of elevated PTS is addressed. The variability of measuring PTS, paired with the inability of PTS to accurately locate the morphology of proximal tibial sagittal deformity, has led us to recently establish a novel measurement, the lateral supratubercle angle (LSTA), which allows for quantification of supratubercle proximal tibia sagittal plane deformity.^16^

While LSTA defines the supratubercle component of PTS, the infratubercle region has not previously been characterized. Therefore, we propose that the lateral infratubercle angle (LITA) be used to identify the infratubercle deformity component of PTS. Introduction and utilization of this novel measurement and its technique will allow quantification of angulation in the infratubercle region of sagittal-plane deformity. Used in conjunction, LSTA and LITA will allow surgeons to identify the underlying drivers of elevated PTS, which, in turn, will allow them to address the underlying location-specific deformity.

Therefore, the primary objective of this study was to describe and establish mean values for this novel measurement of sagittal infratubercle proximal tibial morphology, LITA, within a non-ACL tear cohort, a primary ACL tear cohort, and an ACL graft tear cohort on full-length lateral tibia (FLL) radiographs. The secondary objective of this study was to examine the relationship between LITA, LSTA, and full-length PTS in the primary ACL and ACL-graft tear cohorts. We hypothesized that LITA would differ between non-ACL tear and ACL graft tear cohorts, would positively correlate with PTS elevation, and, when used in conjunction with LSTA, would provide comprehensive detail on the location of the deformity leading to PTS elevation.

## Methods

This was a retrospective study that was approved by the Institutional Review Board at the University of Texas Health Science Center, Houston (HSC-MS-24-637). Patients who presented to 1 of 2 orthopaedic surgeons (A.A.M. and W.R.L.) with knee pain and/or an injury between January 2016 and December 2024 were identified and categorized into 1 of 3 groups: non-ACL tear, primary ACL tear, or ACL graft tear. All patients were then screened for FLL radiographs; those without FLL radiographs were excluded. To identify those with FLL radiographs, the Current Procedural Terminology code 77073 was extracted for both surgeons. The inclusion criteria for all 3 cohorts included closed physes, no previous tibial osteotomy, minimal osteoarthritis, and no fractures. Patients seen in clinic for a knee complaint who were not diagnosed with an operative ACL, medial cruciate ligament, posterior cruciate ligament, or lateral cruciate ligament injury were classified as the non-ACL tear cohort. Patients with a primary ACL tear were included in the primary ACL tear cohort, and patients with failed ACL repair or reconstruction were included in the ACL graft tear cohort. All ACL tears were confirmed by magnetic resonance imaging. Furthermore, radiographs in the ACL graft tear cohort were required to have an unaltered visible tibial tubercle superior edge from previous ACL surgery to ensure accurate measurements. After patient categorization, the quality of the radiographs was assessed. Patients with a calibrated FLL radiograph with posterior femoral condyle obliquity <5 mm were included.

Two independent raters (1 orthopaedic surgery resident and 1 medical student [M.K., C.F.]) performed 5 measurements on each eligible FLL radiograph: PTS along the lateral plateau (PTS-L), PTS along the medial plateau (PTS-M), LSTA along the lateral plateau (LSTA-L), LSTA along the medial plateau (LSTA-M), and LITA. Each rater recorded these measurements for 50 radiographs per cohort for a total of 150 radiographs per rater. Both raters underwent reliability testing and were trained by a dual fellowship-trained orthopaedic surgeon (A.M.). The reliability data utilized were from 30 randomly selected patients in the primary ACL-tear cohort. Inter-rater reliability was assessed between the 2 raters, and intrarater reliability was determined across 2 separate days, with at least 24 hours between measurements.

### Medial and Lateral Plateau Identification for PTS and LSTA Measurements

The medial and lateral tibial plateaus were identified and used for measuring PTS-L, PTS-M, LSTA-L, and LSTA-M. The medial plateau was defined as the most anterior and posterior points of the sclerotic portion of the concave plateau. The lateral plateau was defined as the most anterior and posterior points of the sclerotic portion of the horizontal plateau.^16^

### Measurement Techniques for Full-Length PTS

**Figure 1.**
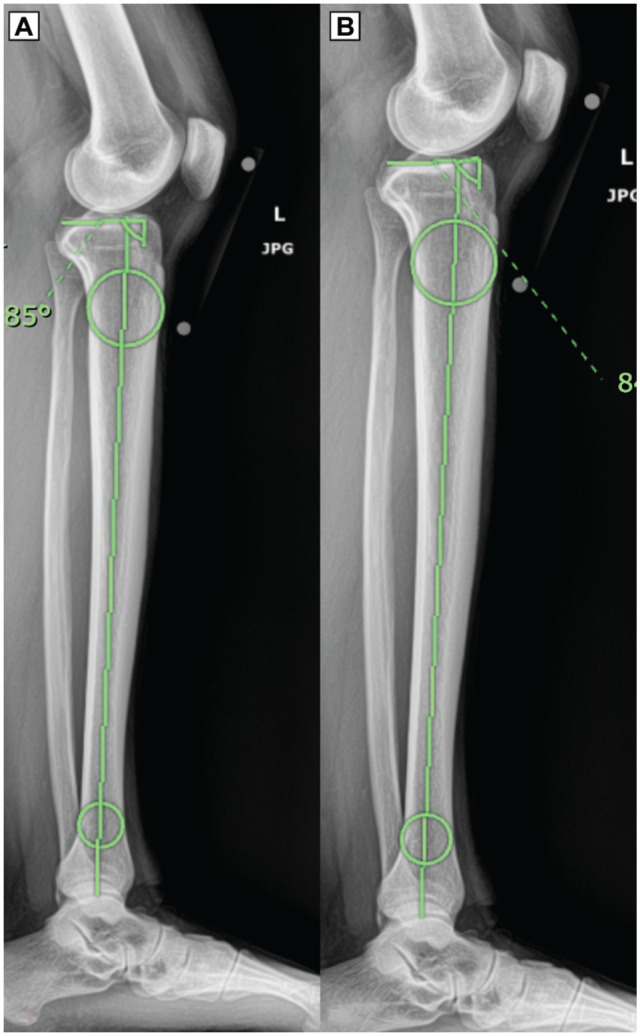
Full-length lateral radiograph measurements of (A) PTS-L and (B) PTS-M. Two circles were created between the anterior and posterior tibial cortices utilizing the method described by Dean et al.^5^ PTS-L, posterior tibial slope-lateral; PTS-M, posterior tibial slope-lateral.

PTS-L and PTS-M were measured on FLL radiographs according to the Dean et al^5^ measurement technique along the anatomical axis. Two circles were created between the anterior and posterior tibial cortices: 1 centered 50 mm distal to the knee, and 1 centered 50 mm proximal to the ankle joint. The midpoints of these circles were connected vertically through the tibia. A second line was drawn along the respective tibial plateau, which was then connected to the vertical line to form a PTS angle (Figure 1). Two PTS measurements were obtained: 1 along the PTS-M and 1 along the PTS-L.

### Measurement Technique for LITA

**Figure 2.**
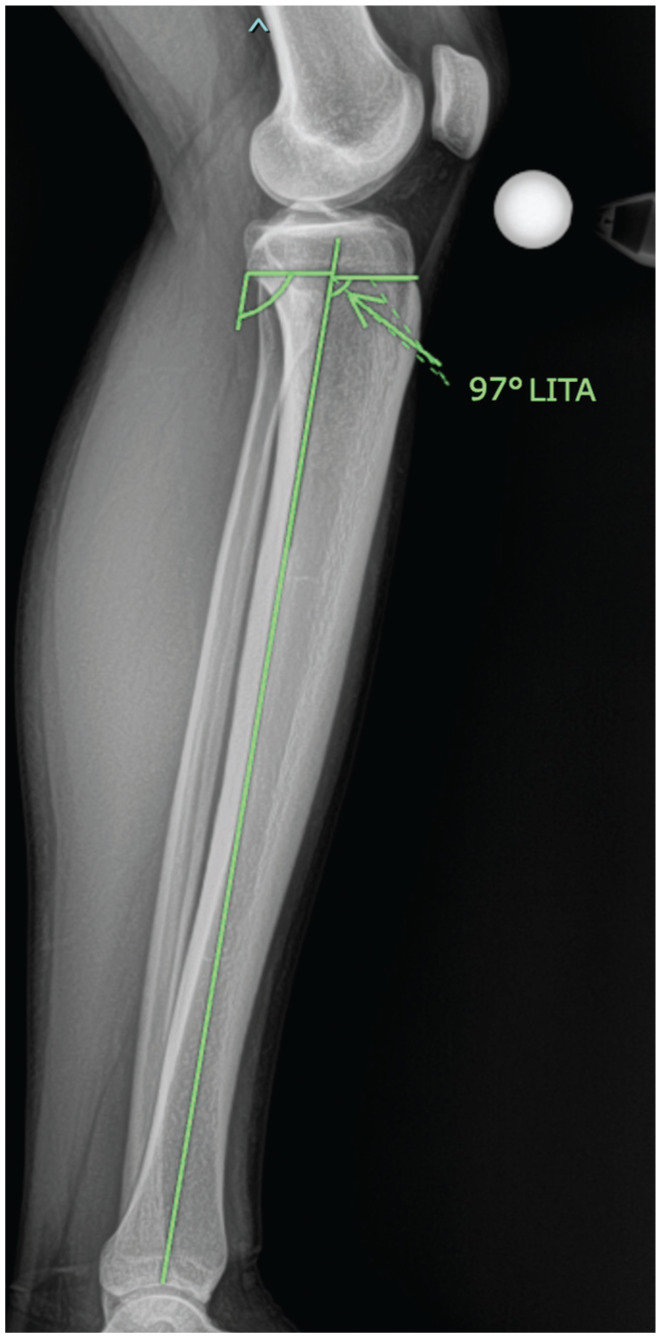
Measurement of LITA on a full-length lateral radiograph using the anterior-facing angle formed at the intersection of a horizontal line connecting the proximal tip of the tibial tubercle and proximal margin of the posterior tibial flare and a vertical line drawn from the center of the tibial plafond to the midpoint of the horizontal line, equating to 97°. LITA, lateral infratubercle angle.

LITA was defined as the anterior-facing angle formed at the intersection of (1) a horizontal line connecting the proximal tip of the tibial tubercle and proximal posterior tibial flare, and (2) a vertical line drawn from the center of the tibial plafond to the midpoint of the horizontal line drawn in step 1 (Figure 2).

### Measurement Technique for LSTA

**Figure 3.**
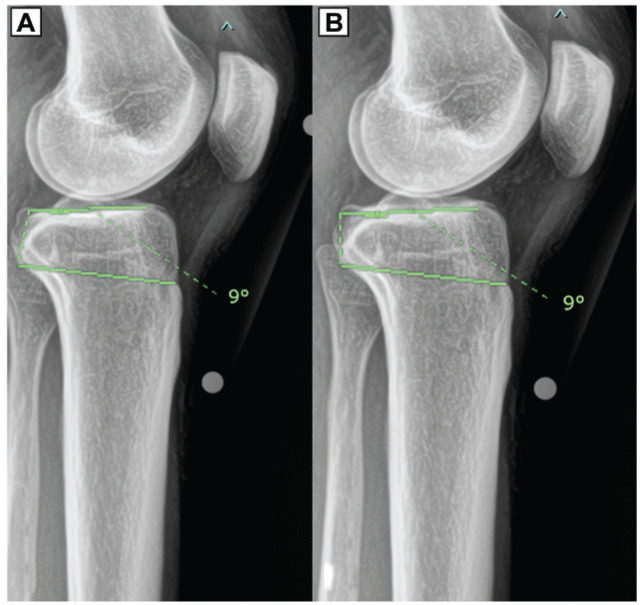
LSTA-L and LSTA-M measurements utilizing the proximal margin of the posterior tibial cortical flare to the tip of the tibial tubercle horizontal line (tubercle-flare line) and respective PL. LSTA-L, lateral supratubercle angle-lateral; LSTA-M, lateral supratubercle angle-medial; PL, plateau lines.

Two measurements of LSTA were obtained: 1 utilizing the medial plateau (LSTA-M) and the other utilizing the lateral plateau (LSTA-L). An angle was created between (1) a line connecting the superior edge of the tibial tubercle to the proximal margin of the posterior cortical flare of the tibia and (2) the line tangent to the medial or lateral plateau, respectively (Figure 3).^16^

### Statistical Analysis

Means, standard deviations, and ranges were calculated for each measurement and cohort. The mean values from the non-ACL tear cohort were used to establish cutoff values for LITA. As this was not a predictive or diagnostic study, cutoff values for LITA were not based on pathological thresholds. Mean values from the non-ACL tear cohort were used to represent an expected morphology of the tibial anatomy in the absence of an ACL injury. Cutoff values for elevated PTS-M and PTS-L were determined from Tollefson et al.^34^ Cutoff values for elevated LSTA-L and LSTA-M were determined from Mansour et al.^16^ To assess whether the data met the assumption of normality, a Shapiro-Wilk test was conducted. In addition to the statistical test, visual inspection of the histograms showed that the data were approximately normally distributed. Therefore, the assumption of normality was considered to be satisfied for parametric analyses.

A series of 1-way analyses of variance (ANOVA) were conducted to compare group differences in PTS-M, PTS-L, LSTA-M, LSTA-L, and LITA values across the 3 cohorts (non-ACL tear, primary ACL tear, and ACL graft tear). To control for multiple comparisons across the 5 dependent variables, a Bonferroni-adjusted significance threshold of *P* < .01 (0.05/5) was applied for comparisons. Eta-squared (η^2^) effect sizes were calculated, with values. A Pearson's correlation coefficient was used to determine the relationship between elevated PTS-M and PTS-L with LSTA-M, LSTA-L, and LITA values across each cohort.

A correlation coefficient of 0.00-0.10 indicated a negligible correlation, 0.10-0.39 a weak correlation, 0.40-0.69 a moderate correlation, 0.70-0.89 a strong correlation, and 0.90-1 a very strong correlation.^27^ Intraclass correlation coefficients (ICCs) were calculated using a 2-way random-effects model with a consistency type to assess the inter-rater and intra-rater reliability for each type of measurement. Interpretation of ICC values followed established guidelines: values <0.5 indicate poor reliability, between 0.5 and 0.75 moderate reliability, between 0.75 and 0.9 good reliability, and >0.9 excellent reliability.^14^ All data were analyzed using the statistical package SPSS Version 28 (IBM Corp). *P* < .05 was used to indicate statistical significance for correlations.

## Results

### Demographic Characteristics

**Figure 4.**
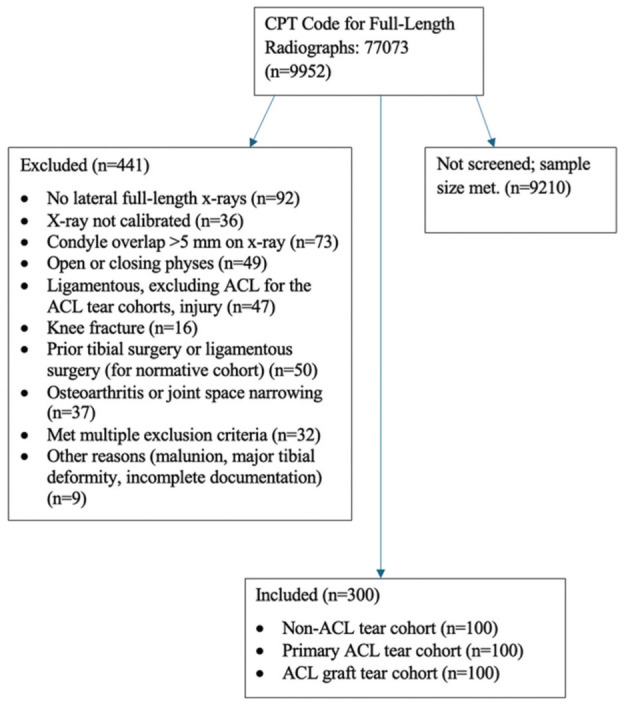
STROBE diagram depicting patient selection criteria for all 3 cohorts. ACL, anterior cruciate ligament; CPT, current procedural terminology. STROBE, Strengthening the Reporting of Observational Studies in Epidemiology.

A total of 100 patients were included in each of the 3 final cohorts for a total of 300 patients (Figure 4). Demographic characteristics of each cohort are shown in Table 1. Included diagnoses of the non-ACL tear cohort are shown in Table 2. There were significant differences in age at the time of radiograph between the non-ACL tear and primary ACL tear cohorts (*P* = .018) and the primary ACL tear and ACL graft tear cohorts (*P* = .015). There was no significant difference in age at the time of radiograph between the non-ACL tear and ACL graft tear cohorts (*P* = .891).

**Table 1:** Demographic Characteristics of the 3 Cohorts*^*a*^*

	Non-ACL Tear Cohort	Primary ACL Tear Cohort	ACL Graft Tear Cohort
Age at time of radiograph, years	30.4 ± 14.4	25.9 ± 12.2	30.1 ± 12.1
Sex, n	50 F, 50 M	53 F, 47 M	48 F, 52 M

a Data are presented as mean ± SD and number of patients. ACL, anterior cruciate ligament; F, female; M, male.

**Table 2:** Table of Included Diagnoses in Non-ACL Tear Cohort*^*a*^*

Included Diagnoses	No.
Osteochondritis dissecans	5
Patellar instability	12
Lateral and/or medial meniscus tears	40
Lateral and/or medial chondral defect	10
Patella and/or patellofemoral chondromalacia	6
Other diagnoses,*^*b*^*	27

a ACL, anterior cruciate ligament.

b Other diagnoses include knee effusion (n = 3); quadriceps tendon rupture (n = 2); discoid meniscus (n = 1); iliotibial band pain (n = 2); lateral cruciate ligament sprain (n = 1); posterior cruciate ligament sprain (n = 1); knee instability (n = 1); patellar tendinopathy (n = 1); lateral femoral condyle osteochondral lesion (n = 1); synovial pathology (n = 3); medial meniscal deficiency (n = 1); lateral or medial compartment syndromes (n = 3); medial femoral condyle injury (n = 2); healthy/no complaint (n = 1); knee derangement s/p meniscus and osteochondral transplants (n = 1); general knee pain (n = 3).

### LITA Descriptives

The mean LITA measurements in the non-ACL tear, primary ACL tear, and ACL graft tear cohorts were 89°± 4.6°, 90.7°± 5.8°, and 92°± 5°, respectively. The mean LITA (89°) of the non-ACL tear group provided a standardized reference point to distinguish between groups; therefore, LITA >89° was classified as elevated.

### Comparisons Between Groups

ANOVA summary statistics, means, standard deviations, and ranges within each cohort for PTS-M, PTS-L, LSTA-M, LSTA-L, and LITA are shown in Table 3. Significant group differences were observed for PTS-M, PTS-L, and LITA. Post hoc Bonferroni tests showed that PTS-M was significantly different between the non-ACL tear and ACL graft tear cohort (*P* < .001). For PTS-L, there were significant differences between the non-ACL tear and ACL graft tear cohorts (*P* < .001), along with the ACL tear and ACL graft tear cohorts (*P* = .002). Regarding LITA, there were significant differences between the non-ACL tear and ACL graft tear cohorts (*P* < .001). There was no significant difference in LITA between the non-ACL tear and primary ACL tear cohorts (*P* = .050). There was no significant difference in LITA between the primary ACL tear and ACL graft tear cohorts (*P* = .171) (Table 3).

**Table 3:** ANOVA Analysis, Mean, Standard Deviations, and Ranges for PTS-M, PTS-L, LITA, LSTA-M, and LSTA-L in the 3 Cohorts*^*a*^*

	Non-ACL Tear	Primary ACL Tear	ACL Graft Tear	ANOVA *P*	*η^2^*
PTS-M, deg	12.2 ± 4 (1-21)	12.7 ± 3.1 (6-20)	14.3 ± 3.5(4-24)	**<.001**	0.065
PTS-L, deg	11.5 ± 3.5 (4-20)	11.8 ± 3.2 (3-18)	13.5 ± 3.6 (6-23)	**<.001**	0.061
LITA, deg	89.0 ± 4.6 (79-104)	90.7 ± 5.8 (75-107)	92 ± 5 (80-108)	**<.001**	0.055
LSTA-M, deg	11.8 ± 4.2 (1-24)	12.3 ± 3.6 (2-26)	11.4 ± 3.4 (2-18)	.272	0.009
LSTA-L, deg	11.1 ± 3.9 (1-20)	11.2 ± 3.8 (3-20)	10.5 ± 3.6 (1-18)	.335	0.007

a Data are reported as mean ± SD (range), unless otherwise specified. Bonferroni-adjusted significance threshold set at *P* < .01. Bold *P* values indicate statistical significance. ACL, anterior cruciate ligament; ANOVA, analysis of variance; LITA, lateral infratubercle angle; LSTA-L, lateral infratubercle angle-lateral plateau; LTSA-M, lateral supratubercle angle-medial plateau; PTS-L, posterior tibial slope-lateral plateau; PTS-M, posterior tibial slope-medial plateau; η^2^, eta squared (effect size).

### Frequencies

Frequencies for patients with elevated PTS (≥12°) and LSTA (>11°) and/or elevated LITA (>89°) within the primary ACL tear and ACL graft tear cohorts are found in Tables 4 and 5. Within the primary ACL tear cohort, 74% (74/100) of patients had either elevated PTS-L and/or PTS-M, and 60.8% (45/74) of them had elevated LITA. Within the ACL graft tear cohort, 86% (86/100) of patients had either elevated PTS-L and/or PTS-M, and 74.4% (64/86) of them had elevated LITA.

**Table 4:** Percentages of Patients With Elevated PTS-M ≥12° and Elevated LITA and/or Elevated LSTA-M*^*a*^*

	Primary ACL Tear With Elevated PTS-M, n = 62	ACL Graft Tear With Elevated PTS-M, n = 82
Normal LSTA-M and elevated LITA	19 (30.7)	35 (42.7)
Normal LITA and elevated LSTA-M	20 (32.3)	20 (24.4)
Elevated LITA and elevated LSTA-M	20 (32.3)	25 (30.5)
Normal LSTA-M and normal LITA	3 (4.8)	2 (2.4)

a Data are presented as n (%). Elevated LITA was defined as >89°; elevated LSTA-M was defined as >11°. ACL, anterior cruciate ligament; LITA, lateral infratubercular angle; LTSA-M, lateral supratubercular angle-medial plateau; PTS-M, posterior tibial slope-medial plateau.

**Table 5:** Percentages of Patients With PTS-L ≥12° and Elevated LITA and/or Elevated LSTA-L*^*a*^*

	Primary ACL Tear With Elevated PTS-L, n = 58	ACL Graft Tear With Elevated PTS-L, n = 70
Normal LSTA-M and elevated LITA	16 (27.6)	33 (47.1)
Normal LITA and elevated LSTA-L	19 (32.8)	13 (18.6)
Elevated LITA and elevated LSTA-L	19 (32.8)	22 (31.4)
Normal LSTA-L and normal LITA	4 (6.9)	2 (2.9)

a Data are presented as n (%). Elevated LITA was defined as >89°; elevated LSTA-L was defined as >11°. ACL, anterior cruciate ligament; LITA, lateral infratubercle angle; LSTA-L, lateral infratubercle angle-lateral plateau; PTS-L, posterior tibial slope-lateral plateau.

### Reliability

A total of 26 patients with an ACL graft tear were included in the analysis. Within-rater reliability was excellent for both raters (>0.914) with narrow 95% CIs. ICCs ranged between good and excellent (0.827-0.989) reliability when analyzing between rater 1 and rater 2 (Table 6). No systematic bias was observed between raters or across repeated measures.

**Table 6:** Intra- and Interrater ICCs in the ACL Graft Tear Cohort*^*a*^*

	Rater 1	Rater 2	Interrater
PTS-M	0.989 (0.974-0.995)	0.981 (0.959-0.992)	0.946 (0.88-0.976)
PTS-L	0.956 (0.901-0.98)	0.971 (0.936-0.987)	0.939 (0.864-0.973)
LITA	0.988 (0.974-0.995)	0.914 (0.809-0.962)	0.959 (0.909-0.982)
LSTA-M	0.971 (0.935-0.987)	0.984 (0.963-0.993)	0.902 (0.782-0.956)
LSTA-L	0.957 (0.901-0.978)	0.965 (0.921-0.984)	0.827 (0.613-0.922)

a Data are presented as the mean measures with ICC (95% CI). ICC, intraclass correlation coefficient. ACL, anterior cruciate ligament; LITA, lateral infratubercle angle; LSTA-L, lateral supratubercle angle–lateral plateau; LSTA-M, lateral supratubercle angle-medial plateau; PTS-L, posterior tibial slope-lateral plateau; PTS-M, posterior tibial slope-medial plateau.

### Correlations

Statistically significant weak to moderate positive correlations were found between PTS-M and LITA in the non-ACL tear cohort (*r*(54) = .337; *P* = .011), the primary ACL tear cohort (*r*(62) = .392; *P* = .002), and the ACL graft tear cohort (*r*(82) = –.475; *P* < .001). Furthermore, there were significant weak to moderate positive correlations between elevated PTS-L and LITA in the non-ACL tear cohort (*r*(49) = .406; *P* = .004), primary ACL tear cohort (*r*(58) = .288; *P* = .029), and ACL graft tear cohort (*r*(70) = .406; *P* < .001) (Table 7). There were no statistically significant differences between PTS-M/L and LSTA-M/L (Table 7).

**Table 7:** Pearson’s Correlation Coefficients in Patients With Elevated PTS ≥12°*^*a*^*

	Non-ACL Tear	Primary ACL Tear	ACL Graft Tear
Elevated PTS-M and LITA	0.337, ***P* = .011**	0.392, ***P* = .002**	0.475, ***P*< .001**
Elevated PTS-L and LITA	0.406, ***P* = .004**	0.288, ***P* = .029**	0.406, ***P* < .001**
Elevated PTS-M and LSTA-M	0.149, *P* = .273	–0.154, *P* = .233	0.164, *P* = .140
Elevated PTS-L and LSTA-L	0.020, *P* = .891	0.053, *P* = .694	0.206, *P* = .087

a Bold *P* values indicate statistical significance (*P* < .05). ACL, anterior cruciate ligament; LITA, lateral infratubercle angle; LSTA-L, lateral infratubercle angle-lateral plateau; LTSA-M, lateral supratubercle angle-medial plateau; PTS-L, posterior tibial slope-lateral plateau; PTS-M, posterior tibial slope-medial plateau.

## Discussion

The most important finding of this study was the introduction and establishment of the LITA in 3 different cohorts: non-ACL tear, primary ACL tear, and ACL graft tear. In the non-ACL tear cohort, we identified a mean LITA angle of 89°, with a range of 79°-104°. Furthermore, the results demonstrated that the mean LITA in the ACL graft tear cohort was significantly higher than in the non-ACL tear cohort, with a 3° increase in LITA from the non-ACL tear to the ACL graft tear cohort.

While the existing literature demonstrates the success of SRO-HTO in correcting elevated PTS, using PTS alone does not allow surgeons to adequately identify the region-specific origins of proximal sagittal tibial deformity.^7,9,12,13,19,20,22,25,28,32^ Because of this, there is no consensus on the ideal osteotomy level relative to the tibial tubercle.^24^ Many SRO-HTOs have previously been performed at the supratubercle level because of the widespread popularity of the technique, which was first described by Dejour and Dejour.^6^ However, SRO-HTO can also be performed at the level of the tubercle or distal to the tubercle.^22,23^ Traditional limb deformity principles recommend that a corrective osteotomy be performed as close as possible to the Center of Rotation of Angulation to minimize the development of secondary deformity.^24^ While each of these surgical techniques has its own unique risks and complications that should be taken into consideration, limb deformity principles suggest that the primary location contributing to the proximal sagittal deformity should drive the osteotomy technique, rather than surgeon preference. To address this gap in locating the region-specific origins of deformity that causes elevated PTS, we recently established the LSTA, which is the angle between the tibial plateau and a line connecting the proximal margin of the posterior tibial cortical flare and the superior edge of the tibial tubercle.^16^ Additionally, Demey et al^8^ established tibial metaphyseal anterior inclination, to also quantify any supratubercle deformity present. While LSTA and tibial metaphyseal anterior inclination aim to identify the presence of supratubercle deformity, they cannot identify any infratubercle deformity that may be present.

Therefore, we have now established the LITA to quantify and localize any deformity in the region distal to the tip of the tibial tubercle. Within the ACL graft tear cohort, our results suggest that, in those with elevated PTS, the infratubercle region may be an important contributor to the deformity. This notion is supported by 2 findings. First, of those with elevated PTS-L or PTS-M in the ACL graft tear cohorts, regardless of LSTA elevation status, 74.4% (64/86) of those patients also had elevated LITA (>89°). Second, there were moderate but significant positive correlations between LITA and elevated PTS-M (*r* = 0.475) and between LITA and elevated PTS-L (*r* = 0.406) in the ACL graft tear cohort, whereas no significant correlations were observed between PTS and LSTA. These results suggest that in an ACL graft tear cohort, patients with elevated PTS may commonly present with an infratubercle deformity component, which should be taken into consideration when surgical planning. We propose using PTS, LSTA, and LITA in conjunction to both identify the presence of sagittal tibial deformity and locate its origins.

Similar to the ACL graft tear cohort, the frequency of elevated LITA was high in the primary ACL tear group, at 61.7% in patients with elevated PTS. Additionally, there were weak but significant positive correlations between elevated PTS-M and LITA (*r* = 0.392) and between elevated PTS-L and LITA (r = 0.288). While these results indicate that an infratubercle contribution to the deformity in the primary ACL tear cohort is common, approximately 32% of patients in this cohort with elevated PTS had only elevated LSTA and normal LITA. This indicates that about one-third of this cohort had primarily a supratubercle deformity. Furthermore, in both the primary ACL tear and ACL graft tear cohorts, 29% to 33% of patients with elevated PTS had both elevated LITA and elevated LSTA, indicating multiple origins of proximal sagittal deformity. Conversely, Demey et al^8^ demonstrated weak to moderate correlations (*r* = 0.362-0.536) between metaphyseal anterior inclination and PTS in patients with a primary ACL tear, and they concluded that the supratubercle region is a larger contributor to PTS deformity. However, their study did not evaluate other potential deformity regions, such as the infratubercle. Our results, paired with those of Demey et al,^8^ emphasize that each elevated PTS is unique and underscores the importance of further evaluating and characterizing both the infratubercle (LITA) and supratubercle (LSTA) components of elevated PTS.

Our data demonstrated that in patients with elevated PTS, the deformity can originate proximal to the tubercle (supratubercle), distal to the tubercle (infratubercle), or be caused by a combination of deformity in both regions. Regarding the clinical applicability of these findings, surgeons can use LITA and LSTA in combination with PTS to provide a region-specific assessment of proximal sagittal tibial deformity and to guide surgical planning. When measuring PTS, LSTA, and LITA, we recommend using full-length lateral radiographs, as these are not interchangeable with standard short-knee radiographs and have been shown to more accurately measure PTS than short-knee radiographs.^10,17^ While PTS and LSTA can be measured on standard short knee radiographs, LITA cannot. As such, using a full-length lateral radiograph allows the same image to be used for all measurements, thereby limiting radiographic variability and enabling region-specific PTS assessment.

Consistent with traditional limb deformity correction philosophy, when infratubercle deformity is determined to be the main driver of elevated PTS, as demonstrated by elevated LITA and normal LSTA, we suggest an infratubercle HTO-SRO to address the proximal sagittal tibial deformity and minimize the potential creation of a secondary deformity. Similarly, when supratubercle deformity is determined to be the primary driver of elevated PTS, demonstrated by elevated LSTA and normal LITA, we suggest that a supratubercle SRO-HTO will address the primary deformity. When patients with elevated PTS present with both elevated LITA and elevated LSTA, we recommend performing the osteotomy at the level of the larger deformity.

### Limitations

There are several limitations of this study that must be acknowledged, the first of which is its retrospective nature. Secondly, although patients were screened in no particular order, there is a potential for selection bias. Additionally, this study did not show significant differences in LSTA between the 3 cohorts. While this conflicts with our previous results, it can be attributed to the smaller sample size of this study compared with the previous study.^16^ However, we acknowledge that a range of normal values exists, and therefore this study should be interpreted in the context of mean values in the 3 separate cohorts. Another limitation is the significant age difference at the time of radiograph between groups. Although a potential confounder, all patients were skeletally mature and without significant arthritis, thereby minimizing the likelihood that age differences would impact PTS and LITA measurements. Lastly, this study was the first step toward quantifying the proximal sagittal tibial deformity in the infratubercle region. It is intended that these results lay the groundwork for future studies to identify the predictive value of LITA and LSTA for ACL retears; as such, the generalizability and potential predictive value of these results cannot yet be determined.

## Conclusion

LITA is a novel measurement that uses distinct anatomic landmarks to quantify infratubercle deformity and can be easily incorporated into FLL radiographic assessment. Elevated LITA was present in most patients with elevated PTS in both the primary ACL tear and ACL graft tear cohorts, indicating a contribution from infratubercle deformity. We recommend utilizing LITA and LSTA with PTS to quantify region-specific proximal sagittal deformity. Future studies should determine whether this quantification can aid surgeons in determining the SRO-HTO technique and influence outcomes.
